# Supplementary material for: Standardizing RNA-seq Analysis of Fungal Pathogens Using BRC-Analytics and Agentic AI: A Candidozyma auris Case Study
Source: bioRxiv. 2025 Dec 30:2025.12.30.697050. Preprint. [Version 1] doi: 10.64898/2025.12.30.697050 (PMC12776552; doi:10.64898/2025.12.30.697050)
Supplement: 1 [file NIHPP2025.12.30.697050V1-supplement-1.pdf]

bioRxiv preprint doi: https://doi.org/10.64898/2025.12.30.697050; this version posted December 31, 2025. The copyright holder for this preprint (which was not certified by peer review) is the author/funder, who has granted bioRxiv a license to display the preprint in perpetuity. It is made available under aCC-BY 4.0 International license.

Supplementary Materials

Supplementary Table 1: *C. auris* WGS data contributors by organization category and top sequencing centers.

bioRxiv preprint doi: https://doi.org/10.64898/2025.12.30.697050; this version posted December 31, 2025. The copyright holder for this preprint (which was not certified by peer review) is the author/funder, who has granted bioRxiv a license to display the preprint in perpetuity. It is made available under aCC-BY 4.0 International license.

Panel A: Summary by Organization Category

Category	Organizations	Runs	% of Total
US State/Local Public Health Labs	26	20,552	78.4%
CDC	2	2,626	10.0%
Academic/Research	46	1,365	5.2%
Other	41	1,345	5.1%
International Public Health	5	313	1.2%
**TOTAL**	**120**	**26,201**	**100%**

Panel B: Top 15 Contributing Organizations

Organization	Full Name	Runs	%
UPHL_ID	Utah Public Health Laboratory	4,447	17.0%
NVSPHL	Nevada State Public Health Laboratory	4,363	16.7%
CDC-NCEZID-MDB	CDC Mycotic Diseases Branch	2,406	9.2%
MDH_CSL	Maryland Dept of Health, Central Services Lab	2,309	8.8%
TXDSHS	Texas Dept of State Health Services	1,487	5.7%
MDHHS-GS	Michigan Dept of Health & Human Services	1,289	4.9%
-	Wisconsin State Laboratory of Hygiene	1,211	4.6%
RIPHL	Rhode Island Public Health Laboratory	1,197	4.6%
NSPHL	Nevada State Public Health Laboratory	1,031	3.9%
-	Wadsworth Center (New York)	705	2.7%
-	Minnesota Dept of Health	688	2.6%
OCPHL_CA	Orange County Public Health Lab (California)	659	2.5%
-	Washington State Dept of Health	583	2.2%
UNLV NPM	Univ of Nevada Las Vegas, Pathogen Monitoring	443	1.7%
-	Fudan University	264	1.0%

US public health laboratories (state/local + CDC) account for 88.4% of all C. auris WGS data, reflecting outbreak surveillance priorities. Nevada appears twice (NVSPHL + NSPHL = 5,394 runs, 20.6%), indicating major outbreak focus.

Supplementary Table 2: RNA-seq methodology across 20 published *C. auris* BioProjects with linked publications (2018-2025).

bioRxiv preprint doi: https://doi.org/10.64898/2025.12.30.697050; this version posted December 31, 2025. The copyright holder for this preprint (which was not certified by peer review) is the author/funder, who has granted bioRxiv a license to display the preprint in perpetuity. It is made available under aCC-BY 4.0 International license.

BioProject	PMID	Authors	Year	Runs	Reference Genome	RNA-seq Tools
PRJNA445471	30559369	Muñoz JF et al.	2018	24	B8441, B11220, B11243	Bowtie2, TopHat2, RSEM, Trinity, edgeR
PRJNA477447	29997121	Kean R et al.	2018	22	B8441 (de novo)	Trinity, HISAT2, Kallisto, DESeq2
PRJNA682185	34630944	Zamith-Miranda D et al.	2021	36	B8441 (GCA_002759435.2)	DESeq2, edgeR
PRJNA682422	34180774	Lara-Aguilar V et al.	2021	6	B8441 (GCA_002759435.2)	FastQC, Trimmomatic, fastp, STAR, featureCounts, DESeq2
PRJNA735406	34354695	Zhou W et al.	2021	6	B11221 (Clades I-V)	Trimmomatic, HISAT2, Cufflinks, HTSeq, DESeq2
PRJNA801628	35473297	Biermann AR et al.	2022	24	B8441, B11221, B11243 (Clades I, III, IV)	HISAT2, featureCounts, edgeR
PRJNA830685	36445083	Narayanan A et al.	2022	16	B8441, CBS10913 (Clade II)	FastQC, fastp, BWA, Bowtie2, HTSeq, DESeq2
PRJNA788930	35652307	Shivarathri R et al.	2022	12	NS	RNA-seq
PRJNA792028	36913408	Bing J et al.	2023	15	GCA_002759435.2, GCF_002775015.1	HiSat2, StringTie, DESeq2, BWA
PRJNA904261	37769084	Santana DJ et al.	2023	6	B8441 (Clade I)	RNA-seq
PRJNA1015296	38493178	Bing J et al.	2024	141	B8441 (GCA_002759435.2)	HiSat2, StringTie, DESeq2, BWA
PRJNA902676	38722168	Yang B et al.	2024	40	B11220, B11221 (Clades II, III)	Kallisto, DESeq2
PRJNA1036037	39480072	Li J et al.	2024	22	Clade IV	RNA-seq
PRJNA1086003	39455573	Wang TW et al.	2024	13	B8441 (Clade I)	HISAT2, STAR, DESeq2
PRJEB57846	39297640	Rhodes J et al.	2024	12	NS	WGS, RNA-seq
PRJNA1012821	40468551	Chauhan A et al.	2025	16	B8441, B11220 (CGD)	FastQC, fastp, Bowtie2, HTSeq, DESeq2
PRJNA1139166	40099908	Phan-Canh T et al.	2025	15	B8441 (GCA_002759435.2)	FastQC, fastp, cutadapt, STAR, featureCounts
PRJNA1208975	40530673	Yang G et al.	2025	9	Clade I	RNA-seq
PRJNA1232830	40066990	Chauhan M et al.	2025	6	Clade I	RNA-seq

bioRxiv preprint doi: https://doi.org/10.64898/2025.12.30.697050; this version posted December 31, 2025. The copyright holder for this preprint (which was not certified by peer review) is the author/funder, who has granted bioRxiv a license to display the preprint in perpetuity. It is made available under aCC-BY 4.0 International license.

BioProject	PMID	Authors	Year	Runs	Reference Genome	RNA-seq Tools
PRJNA1291775	40863525	Vidal-Montiel A et al.	2025	6	GCA_003014415.1, GCA_034640365.1 (Clades III, IV)	FastQC, Trimmomatic, STAR, featureCounts, DESeq2

NS = Not specified in available text. Data extracted from PMC full text via EuropePMC and NCBI E-utilities APIs. 44 additional BioProjects had no linked publications.

Supplement 1

A "plan" generated by Claude Code:

Goal: Split collection #244 into 3 condition-specific collections for DESeq2.

Experimental conditions (from paper):

Condition	Description	Replicates
AR0382_WT	Clade I reference, highly adhesive	2 (A, B)
AR0387_WT	Clade I, poorly adhesive (206 SNPs from AR0382)	2 (A, B)
tnSWI1	SWI1 insertional mutant of AR0382	2 (A, B)

Expected SRR mapping (to verify): - AR0382_WT: SRR22376031, SRR22376032 - AR0387_WT: SRR22376029, SRR22376030 - tnSWI1: SRR22376027, SRR22376028

Approach (from galaxy-agentic-collection-transform): 1. Fetch SRA metadata → confirm mapping 2. Check collection #244 element naming 3. Create identifier files for each condition 4. Use **FILTER_FROM_FILE** Galaxy tool to split 5. Rename output collections

Next steps before execution: 1. Verify SRA metadata 2. Check collection #244 element names 3. Present findings for your approval
